# *Carpesium abrotanoides* ethanol extract alleviates dextran sulfate sodium-induced ulcerative colitis by suppressing inflammation and apoptosis

**DOI:** 10.3389/fphar.2025.1672111

**Published:** 2025-09-29

**Authors:** Aejin Kim, So Yeon Kim, Kyuhyung Jo, Eunjung Son, Chan-Sik Kim, Dong-Seon Kim, Youn-Hwan Hwang, Yun Mi Lee

**Affiliations:** ^1^ KM Convergence Research Division, Korea Institute of Oriental Medicine, Daejeon, Republic of Korea; ^2^ Korean Convergence Medical Science Major, Campus of Korea Institute of Oriental Medicine, University of Science & Technology, Daejeon, Republic of Korea; ^3^ KM Science Research Division, Korea Institute of Oriental Medicine, Daejeon, Republic of Korea

**Keywords:** *Carpesium abrotanoides*, ulcerative colitis, network pharmacology, inflammation, apoptosis, tight junction

## Abstract

**Background:**

*Carpesium abrotanoides* has been traditionally used to treat various inflammatory and infectious diseases. However, there is no scientific report on its protective activity against intestinal inflammatory disorders, including ulcerative colitis (UC). In this study, we aimed to investigate the mechanisms underlying the protective effects of *C. abrotanoides* extract (CAE) in UC treatment.

**Materials and Methods:**

Key components of CAE were identified through ultra-performance liquidchromatography, and their potential targets and pathways were predicted through network pharmacology and molecular docking. The therapeutic effects of CAE were evaluated in a dextran sulfate sodium-induced UC mouse model by assessing clinical parameters, colon length, histopathology, and the expression of inflammatory, tight junction, and apoptosis-related markers.

**Results:**

The components of CAE, including chlorogenic acid, kaempferol 3-O-rhamnoside, 1,3-dicaffeoylquinic acid, 3,5-dicaffeoylquinic acid, and 4,5-dicaffeoylquinic acid, were identified. These components interacted with critical targets, including tumor necrosis factor, interleukin-6, interleukin-1β, caspase-3, and Bcl-2, modulating inflammatory and apoptotic pathways. *In vivo* experiments showed that CAE reduced the disease activity index, prevented colon shortening, and ameliorated histological damage. It preserved tight junction proteins (ZO-1 and claudin-1), reduced inflammatory cell infiltration, and downregulated pro-inflammatory mediators. Moreover, CAE inhibited the expression of the pro-apoptotic protein Bax, the execution-phase apoptotic markers cleaved caspase-3 and cleaved PARP, while upregulated the expression of the anti-apoptotic protein Bcl-2.

**Conclusion:**

CAE alleviates dextran sulfate sodium-induced colitis by exerting anti-inflammatory and anti-apoptotic effects and maintaining intestinal barrier integrity. These findings support the potential of CAE as a natural multitarget therapeutic agent for UC.

## 1 Introduction

Inflammatory bowel disease (IBD), most notably Crohn’s disease and ulcerative colitis (UC), is a chronic inflammatory disorder of the gastrointestinal tract. It is characterized by persistent mucosal inflammation and tissue injury in the small and large intestines, accompanied by clinical symptoms, including abdominal pain, diarrhea, and rectal bleeding. UC affects approximately 5 million people, with prevalence and incidence continuing to rise steadily worldwide (Le Berre et al., 2023). The exact cause of the disease remains unclear; however, a complex interplay of autoimmune responses, genetic predisposition, alterations in the gut microbiota, and environmental factors is believed to be responsible (Porter et al., 2020; Qiu et al., 2025; Zhao et al., 2023). In UC, an abnormal immune response to the intestinal microbiota triggers the release of pro-inflammatory cytokines and the infiltration of immune cells into the colonic mucosa. This damages the structure and function of tight junctions (TJ), increases intestinal permeability, and enables the passage of luminal antigens through the barrier to interact with the immune cells in the intestinal mucosa. This interaction activates immune pathways, secretes proinflammatory cytokines, and infiltrates inflammatory cells, creating a continuous vicious cycle of tissue damage and inflammation. Chronic UC may lead to serious complications, including colon cancer, strictures, and an increased risk of infection, thereby exacerbating systemic inflammation, which can significantly reduce a patient’s quality of life (Chang et al., 2024).

Current treatments for UC focus on controlling inflammation, including mesalamine, corticosteroids, immunomodulators, biologics, and small molecule drugs that suppress excessive inflammatory responses. Mesalamine is considered afirst-line maintenance therapies, the remission rate is about 50%. However, these drugs cannot completely cure UC, are associated with a risk of relapse, often have reduced efficacy, and cause various side effects when used long-term (D'Haens, 2007; van der Lelie et al., 2021; Zeng et al., 2022). Recently, TJ dysfunction has been closely associated with the severity, activity, and recurrence of IBD in patients. Therefore, in treating UC, it is important to suppress inflammation and repair damaged intestinal mucosa. Hence, multifaceted mechanistic studies are required (Kuo et al., 2021; Villanacci et al., 2025; Weber et al., 2008).

For these reasons, natural products are now considered promising therapeutic alternatives owing to their multicomponent properties and ability to simultaneously modulate multiple biological targets. Additionally, these products have low toxicity and are suitable for long-term use, which supports their potential as therapeutic agents for UC (Li et al., 2024; Li et al., 2022; Liang et al., 2025). Network pharmacology is a systems biology approach in natural product research that enables the prediction and analysis of multi-target mechanisms by integrating chemical, pharmacological, and systems biology data (Noor et al., 2023; Zhai et al., 2025).

*Carpesium abrotanoides*, a member of the Asteraceae family, has been traditionally used in East Asian medicine to treat various inflammatory and infectious diseases, including sore throat, bronchitis, acute hepatitis, expelling worms, tonsillitis, malaria, and itchy skin (Fang and Zhao, 2007). Phytochemical analyses have been used to identify sesquiterpene lactones, flavonoids, and triterpenoids as major bioactive constituents. Reportedly, the extracts and compounds isolated from *C. abrotanoides* can exhibit anti-inflammatory, antioxidant, anti-tumor, anti-plasmodial, anti-fungal, anti-bacterial, and cytotoxic activities, suggesting their potential as a source of therapeutic agents. Furthermore, the aerial extract demonstrated antioxidant potential in various assays, including ABTS (2,2′-azinobis-3-ethylbenzothiazoline-6-sulfonic acid), DPPH (1,1-diphenyl-2-picrylhydrazyl), NOS (nitric oxide scavenging), and FIC (ferrous ion chelating). It also modulated cyclooxygenase-2 (COX-2) expression induced by various Toll-like receptor (TLR) agonists in mouse macrophages, demonstrating its efficacy as a therapeutic agent for chronic inflammatory diseases (Ibrahim et al., 2022; Liu X. et al., 2023; Liu Z. et al., 2023; Zhang et al., 2015). Although various studies have examined the anti-inflammatory properties of CAE, investigations specifically addressing its therapeutic effects on UC remain unexplored.

Therefore, in this study, we identified the main compounds in *C. abrotanoides* extract (CAE) and utilized network pharmacology to predict their mechanisms of action against UC. Furthermore, the therapeutic potential of CAE was validated in a dextran sulfate sodium (DSS)-induced colitis mouse model, confirming its efficacy and involvement in key pathways predicted by network pharmacology analysis. These findings offer evidence supporting the potential of CAE as a functional food ingredient or therapeutic agent for UC.

## 2 Materials and methods

### 2.1 Preparation of CAE

In July 2022, samples of *C. abrotanoides* were obtained from Jeju (South Korea) and identified by Professor Geung-Joo Lee (Chungnam National University, South Korea). A voucher specimen was deposited in the Korean Herbarium of Standard Herbal Resources of the Korea Institute of Oriental Medicine (202401025219, Daejeon, South Korea). Dried *C. abrotanoides* leaves were finely powdered with an electric mill, and 100 g of the sample was extracted with 1.5 L of 70% ethanol for 3 h at reflux. The extract was concentrated under reduced pressure, freeze-dried, and stored at 4 °C (yield: 26.7%).

### 2.2 Analytical conditions of CAE

An ultra performance liquid chromatography (UPLC, Waters, MA, United States) system equipped with a quaternary pump, auto–sampler, and photodiode array detector with UPLC^®^ BEH C18 (150 × 2.1 mm, 1.7 μm particle size), was used for analysis. Elution was performed using solvent A (0.1% phosphoric acid with water) and solvent B (acetonitrile) in an elution gradient at a rate of 0.3 mL/min as follows: 0–2 min, 5%–5% B; 2–5 min, 5%–25% B; 5–10 min, 25%–25% B; 10–13 min, 40%–100% B; 17–19 min, 100%–5% B; 19–21 min, 5%–5% B. The detection wavelength was 284 nm, the column temperature was maintained at 40 °C, and the injection volume was 2 µL.

### 2.3 Network pharmacology analysis

#### 2.3.1 Prediction of target genes

The structural data file of the target genes and SMILES format were obtained from the PubChem database (https://pubchem.ncbi.nlm.nih.gov/) to predict the potential target genes of the selected compound. Target prediction was performed using SwissTargetPrediction (http://www.swisstargetprediction.ch/), PubChem (https://pubchem.ncbi.nlm.nih.gov/), STITCH (https://stitch.embl.de/), and BATMAN (http://bionet.ncpsb.org.cn/batman-tcm/#/home), based on their chemical structures. UC-related target genes were retrieved from the GeneCards database (https://www.genecards.org/), Online Mendelian Inheritance in Man (https://www.omim.org/), and Therapeutic Target Database (https://idrblab.net/ttd/) using the keyword “ulcerative colitis.” Common targets between the compound and the disease were identified by creating a Venn diagram using Venny 2.1 (https://bioinfogp.cnb.csic.es/tools/venny/).

#### 2.3.2 Protein–protein interaction network

The common target genes were uploaded to the STRING database (https://string-db.org/) to create a protein–protein interaction (PPI) network. “*Homo sapiens”* was used for species, with a confidence score >0.400 (medium confidence). The resulting interaction network was visualized and further analyzed using Cytoscape (v3.9.1), and 10 hub genes were identified based on their degree of centrality using the CytoHubba plugin.

#### 2.3.3 Gene ontology and kyoto encyclopedia of genes and genomes pathway enrichment analysis

Gene Ontology (GO) enrichment analysis, including biological processes, molecular functions, and cellular components, and Kyoto Encyclopedia of Genes and Genomes (KEGG) pathway analysis, was conducted using the STRING platform. GO terms and KEGG pathways were further filtered to include those that (1) contained at least two genes among the top 30 hub genes identified from the PPI network, (2) had a count of five, and (3) showed a false discovery rate (FDR) < 0.01. The top 20 data points were visualized using the R software (Team, 2023).

#### 2.3.4 Molecular docking simulation

The three-dimensional crystal structures of the target proteins were retrieved from the RCSB Protein Data Bank (PDB) (https://www.rcsb.org/), and Open Babel was used to prepare ligand structures. Active binding pockets were predicted using PrankWeb (https://prankweb.cz/) and CASTp (http://sts.bioe.uic.edu/castp/). Molecular docking and binding affinity calculations (kcal/mol) were performed using AutoDock tools 1.5.7 and AutoDock Vina software. Docking conformations were further visualized using Discovery Studio Visualizer (version 25.1.0.24284).

Binding affinities were interpreted based on widely accepted empirical criteria: strong binding interactions were defined as ≤ −9.0 kcal/mol; moderate binding between −7.0 and −9.0 kcal/mol; weak binding between −5.0 and −7.0 kcal/mol; and very weak or nonspecific binding interactions were indicated using values above −5.0 kcal/mol (Dallakyan, 2015; Ferreira et al., 2015; Meng et al., 2011; Zhang et al., 2019).

### 2.4 *In vivo* experimental design

For the *in vivo* experiment, 7-week-old female C57BL/6 mice were obtained from Orient Bio (Seongnam, South Korea). The Institutional Animal Care and Use Committee of the Korea Institute of Oriental Medicine (KIOM-23-079) approved all experiments, which were carried out in accordance with the Guide for the Care and Use of Laboratory Animals published by the US National Institutes of Health (Bethesda, MD, United States). The mice were allowed to acclimate for a 7-day period under standard laboratory conditions with *ad libitum* access to water and food. The mice were divided into five groups (n = 8 per group): vehicle-treated control (Con), 3.5% DSS (DSS), 3.5% DSS + CAE 100 mg/kg (CAE-100), 3.5% DSS + CAE 200 mg/kg (CAE-200), and 3.5% DSS + mesalamine 50 mg/kg (PC). CAE and mesalamine were administered orally for 7 days, followed by the induction of colitis using drinking water containing 3.5% DSS for 7 days, with CAE and mesalamine administration. On day 8, the blood and colon were collected, and the colon length was recorded. Body weight, stool condition, and total blood in the feces were recorded daily to monitor the severity of colitis (Table 1). The disease activity index (DAI) score was calculated as the mean value of body weight loss, stool consistency, and hematochezia scores (Table 1).

**TABLE 1:** Scoring of the disease activity index.

Score	Weight loss	Stool consistency	Visible blood in feces
0	None	Normal	None
1	1%–5%	Soft stool but still formed	Positive hemoccult
2	6%–10%	Loose stool	Observable traces of blood in stool
3	11%–20%	Mild diarrhea	Mild bleeding
4	>20%	Severe diarrhea	Gross bleeding

### 2.5 Measurement of cytokines

Serum IL-6 and IL-1β levels were determined using an enzyme-linked immunosorbent assay kit (R&D System, MN, United States) following the manufacturer’s protocol.

### 2.6 Real-time quantitative polymerase chain reaction

Total RNA was extracted from colonic tissues in an RNase-free setting using the RNeasy Mini Kit (Cat# 74904; Qiagen), following the manufacturer’s instructions. The PrimeScript RT Master Mix Perfect Real-Time Kit (Cat# R047A; TaKaRa) was used to generate cDNA. Real-time quantitative polymerase chain reaction (PCR) was performed using a QuantStudio 3 Real-Time PCR System (Applied Biosystems) with the SYBR Green SYBR Premix Ex Taq kit (Cat# RR820A, TaKaRa). The gene expression levels were measured using the ^ΔΔ^Ct method, and β-actin served as the internal control for normalization. The primer sequences are listed in Table 2.

**TABLE 2:** Primer sequences that were used for the mRNA expression analyses.

Gene		Primer sequence
*Il1b*	Forward	AGT​GCA​GCT​GTC​TAA​TGG​GA
	Reverse	GCC​CAT​CCT​CTG​TGA​CTC​A
*Tnf*	Forward	GCAGAGAGGTTGACT TTC
	Reverse	CTA​CTC​CCA​GGT​TCT​CTT​CAA
*Il-6*	Forward	TCC​AGT​TGC​CTT​CTT​GGG​AC
	Reverse	GTG​TAA​TTA​AGC​CTC​CGA​CTT​G
*Ptgs2*	Forward	ACT​CAC​TCA​GTT​TGT​TGA​GTC​ATT​C
	Reverse	TTT​GAT​TAG​TAC​TGT​AGG​GTT​AAT​G
*Nos2*	Forward	GAG​ACA​GGG​AAG​TCT​GAA​GCA​C
	Reverse	CCA​GCA​GTA​GTT​GCT​CCT​CTT​C
*Casp3*	Forward	GGA​GTC​TGA​CTG​GAA​AGC​CGA​A
	Reverse	CTT​CTG​GCA​AGC​CAT​CTC​CTC​A
*Parp1*	Forward	CCA​GCG​CAG​CTC​AGA​GAA​GCC​A
	Reverse	CAT​GTT​CGA​TGG​GAA​AGT​CCC
*Bax*	Forward	GCT​GAT​GGC​AAC​TTC​AAC​TG
	Reverse	GAT​CAG​CTC​GGG​CAC​TTT​AG
*Bcl2*	Forward	TCC​TTC​CAG​CCT​GAG​AGC​AAC​C
	Reverse	TCA​CGA​CGG​TAG​CGA​CGA​GAG
*Gapdh*	Forward	5′- CAT​ACC​AGG​AAA​TGA​GCT​TG-3′
	Reverse	5′- ATG​ACA​TCA​AGA​AGG​TGG​TG-3′

### 2.7 Western blotting

Total protein was extracted from the cells using a radioimmunoprecipitation assay buffer. Approximately 15 μg of protein was separated using 10% sodium dodecyl sulfate-polyacrylamide gel electrophoresis and subsequently transferred onto polyvinylidene difluoride membranes (Millipore, Burlington, MA, United States). The membranes were blocked with 5% non-fat milk in tris-buffered saline containing 0.05% Tween-20 for 1 h and incubated with primary antibodies overnight at 4 °C, followed by horseradish peroxidase-labeled secondary antibodies for 1 h at room temperature. The membranes were subsequently washed with 0.05% Tween-20, and signals were detected using an image analyzer (LAS 4000 mini; GE Healthcare Bio-Sciences, NJ, United States) with an enhanced chemiluminescence solution. β-actin was used as the internal reference, and relative expression was measured using the ImageJ software (National Institutes of Health, Bethesda, MD, United States).

### 2.8 Histological analysis

The dissected tissues were fixed in 10% neutral-buffered formalin for histological analysis and embedded in paraffin. Paraffin-embedded sections were cut into 4 µm-thick sections and stained with hematoxylin and eosin (H&E). Stained images were captured in random microscopic areas from independent animals using an Axioscan7 digital slide scanner (Zeiss, Germany). Histopathological scoring was performed in a blinded manner using a semi-quantitative scale ranging from 0 to 9, with higher scores indicating more severe pathological alterations. The scoring system consisted of three parameters: epithelial damage (0–3), inflammatory cell infiltration (0–3), and crypt damage/ulceration (0–3). The total histopathological score was calculated as the sum of these parameters (Koelink et al., 2018; Zhang et al., 2025).

### 2.9 Immunohistochemistry

The paraffin sections were deparaffinized, hydrated, and treated with 1% hydrogen peroxide in methanol. After antigen retrieval and blocking, all sections were incubated with primary antibodies against caspase-3 (1:500 dilution, Cell Signaling, 9665S, Danvers, MA, United States) and zonula occludens-1 (ZO-1) (1:200 dilution, Abcam, ab96587, San Francisco, CA, United States) at appropriate temperatures and times. An immune response was induced using the ImmPRESS^®^ horseradish peroxidase goat anti-rabbit IgG polymer detection kit peroxidase (Vector Laboratories, MP-7451, Burlingame, CA, United States). A DAB Chromogen/Substrate Kit (High Contrast) (Scytek, UT, United States) was used for visualization, and counterstaining was performed using hematoxylin. Image scanning, analysis, and editing were performed as described in Section 2.8.

### 2.10 Terminal deoxynucleotidyl transferase dUTP nick end labeling staining

The level of apoptosis in the colon sections was determined using terminal deoxynucleotidyl transferase dUTP nick end labeling (TUNEL) staining (Roche, Cat# 11684795910) following the manufacturer’s instructions. The nucleus with 4,6′-Diamidino-2-phenylindole (DAPI; Abcam, Cat#: ab104139) was stained, and the TUNEL-positive cells were counted (a minimum of 10 crypts with normal morphology were counted per section).

### 2.11 Statistical analysis

GraphPad Prism 7 software (GraphPad Software, San Diego, CA, United States) was used to perform all statistical analyses. Data are shown as mean ± standard deviation. Differences between groups were evaluated using one-way ANOVA, followed by Dunnett’s multiple comparison test. Statistical significance was set at P < 0.05.

## 3 Results

### 3.1 Identification of CAE components

**FIGURE 1:**
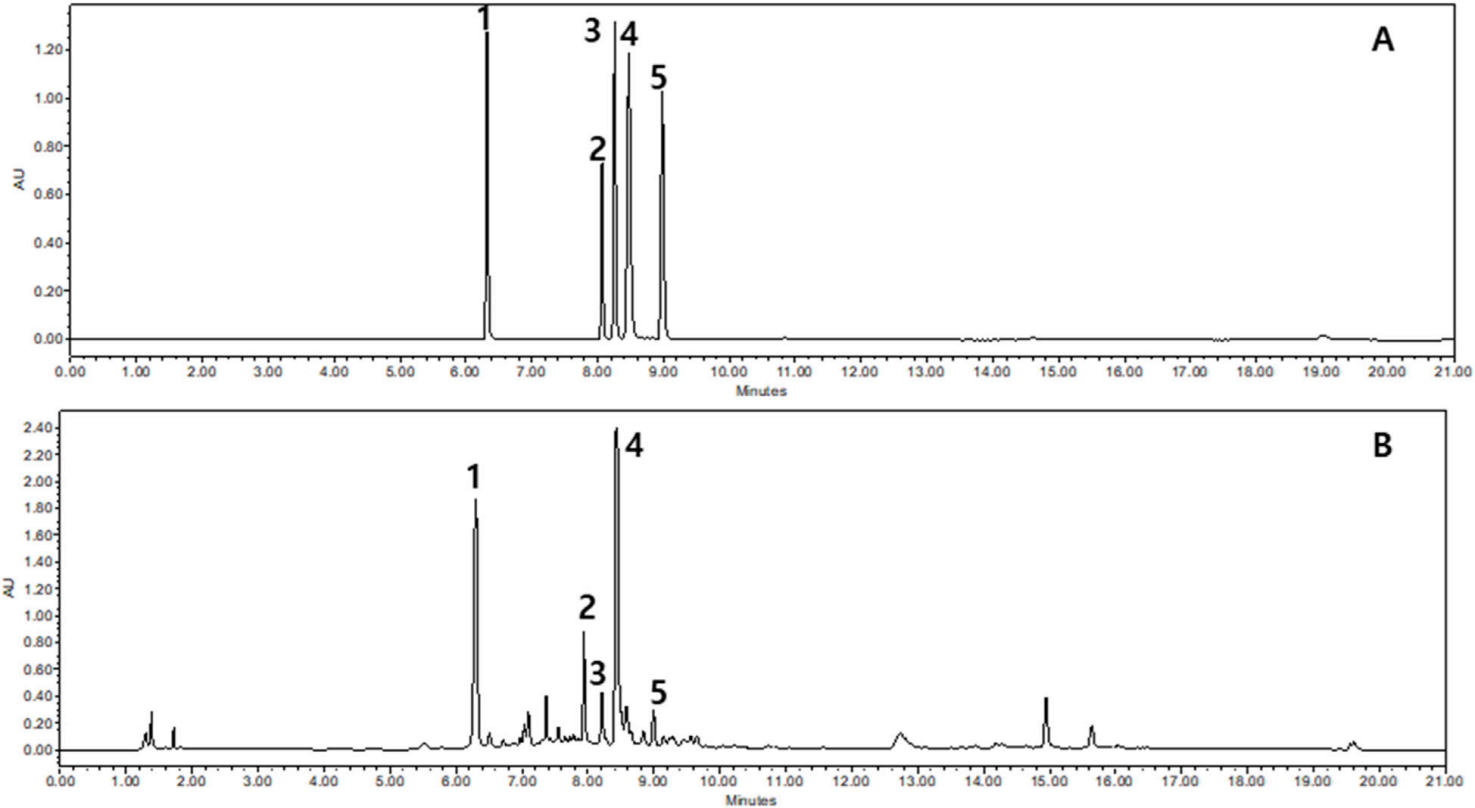
Representative ultra performance liquid chromatography results of Carpesium abrotanoides extract (CAE) at 280 nm: (1) chlorogenic acid; (2) kaempferol 3-O-rhamnoside; (3) 1,3-dicaffeoylquinic acid; (4)3,5-dicaffeoylquinic acid; and (5) 4,5-dicaffeoylquinic acid. **(A)** Mixed standard solution and **(B)** CAE.

CAE was analyzed using UPLC. We identified five phytochemicals predominantly present in the extract by comparing the retention times and mass spectra with those of reference standards (A). The quantity of each compound was 25.1 ± 0.48 mg/g for chlorogenic acid, 10.8 ± 0.09 mg/g for Kaempferol 3-O-rhamnoside, 2.6 ± 0.05 mg/g for 1,3-dicaffeoylquinic acid, 39.5 ± 0.20 mg/g of 3,5-dicaffeoylquinic acid, and 2.2 ± 0.16 mg/g for 4,5-dicaffeoylquinic acid (Figure 1).

### 3.2 Network pharmacology analysis

#### 3.2.1 Prediction of the potential targets for UC by CAE

**FIGURE 2:**
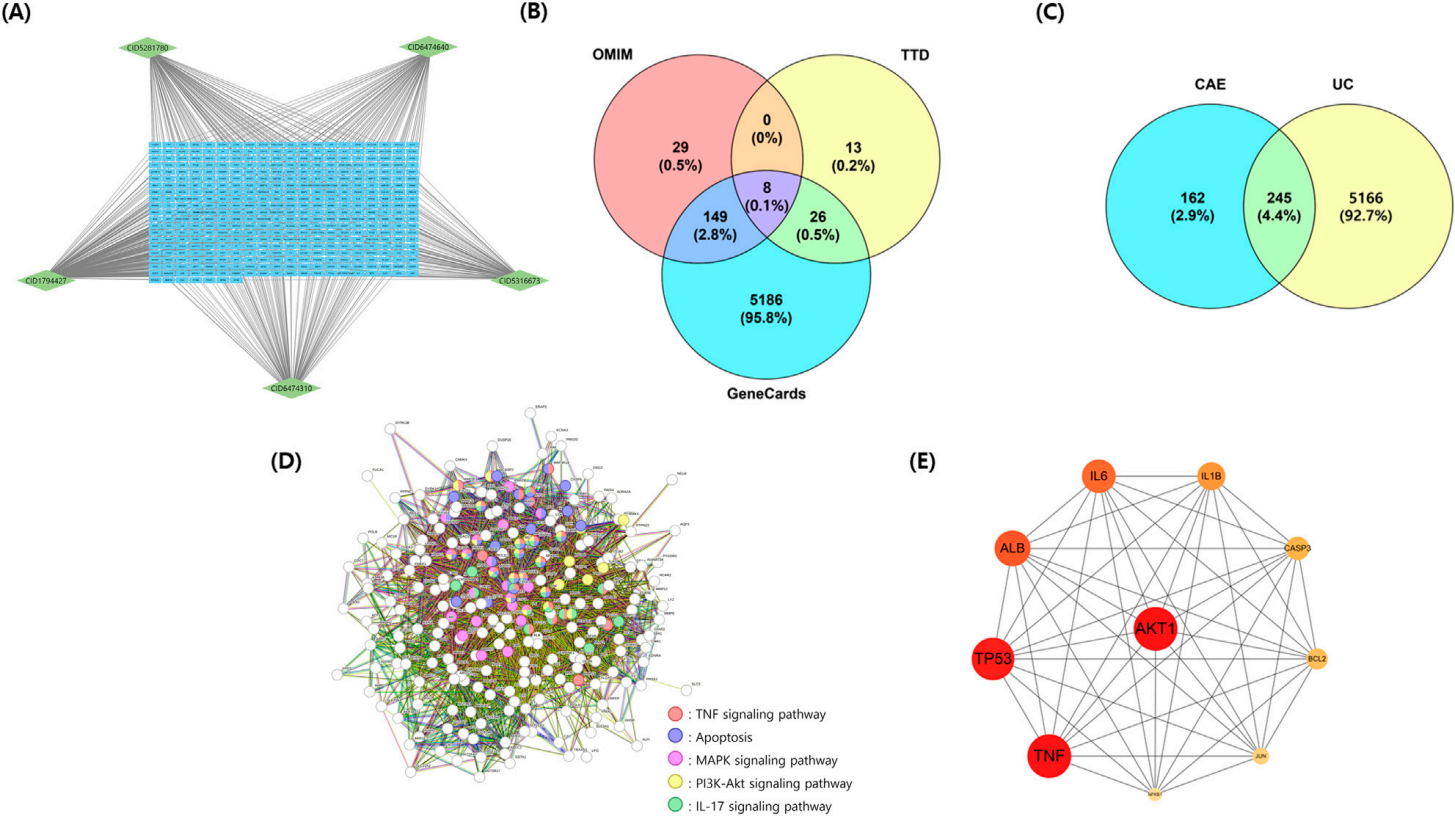
Network construction and analysis of CAE for UC. **(A)** Compound-target network of CAE. **(B)** UC-related target genes identified from GeneCards, OMIM, and TTD. **(C)** Venn diagram showing 245 common target genes. **(D)** Protein–protein interaction network of the common targets generated using STRING. **(E)** Top 10 core genes ranked by degree centrality using CytoHubba. CAE, *Carpesium abrotanoides* extract; OMIM, Online Mendelian Inheritance in Man; TTD, Therapeutic Target Database; UC, ulcerative colitis.

A network pharmacology approach was used to investigate the mechanism of action of CAE in treating UC. The targets of the five CAE components were predicted using PubChem, STITCH, BATMAN, and SwissTarget Prediction. Overall, 407 targets were identified in the database. As the interaction between targets is essential in the interaction of CAE, we analyzed the compound-target network of CAE (Figure 2A). We searched for UC-related genes acquired from Online Mendelian Inheritance in Man, Therapeutic Target Database, and GeneCards to identify UC-associated targets of CAE. As shown in Figure 2B, 5,411 target genes were identified from the three databases after removing duplicate data. Potential targets of CAE for improving UC were screened using Venny 2.1, identifying 245 common genes (Figure 2C).

#### 3.2.2 Analysis of targets in the PPI network

Using the STRING database, protein interaction analysis was performed on 245 common target genes to ameliorate UC from CAE. A PPI network was created (Figure 2D). Overall, 244 nodes and 5,688 edges were obtained. Core target proteins in the PPI network were screened using Cytoscape 3.9.1. The top 10 interactions were identified based on their degree of centrality within the CytoHubba plugin. The results showed that TNF, AKT1, TP53, ALB, IL6, IL1B, CASP3, BCL2, JUN, and NFKB1 were obtained (Figure 2E).

#### 3.2.3 Enrichment analysis

**FIGURE 3:**
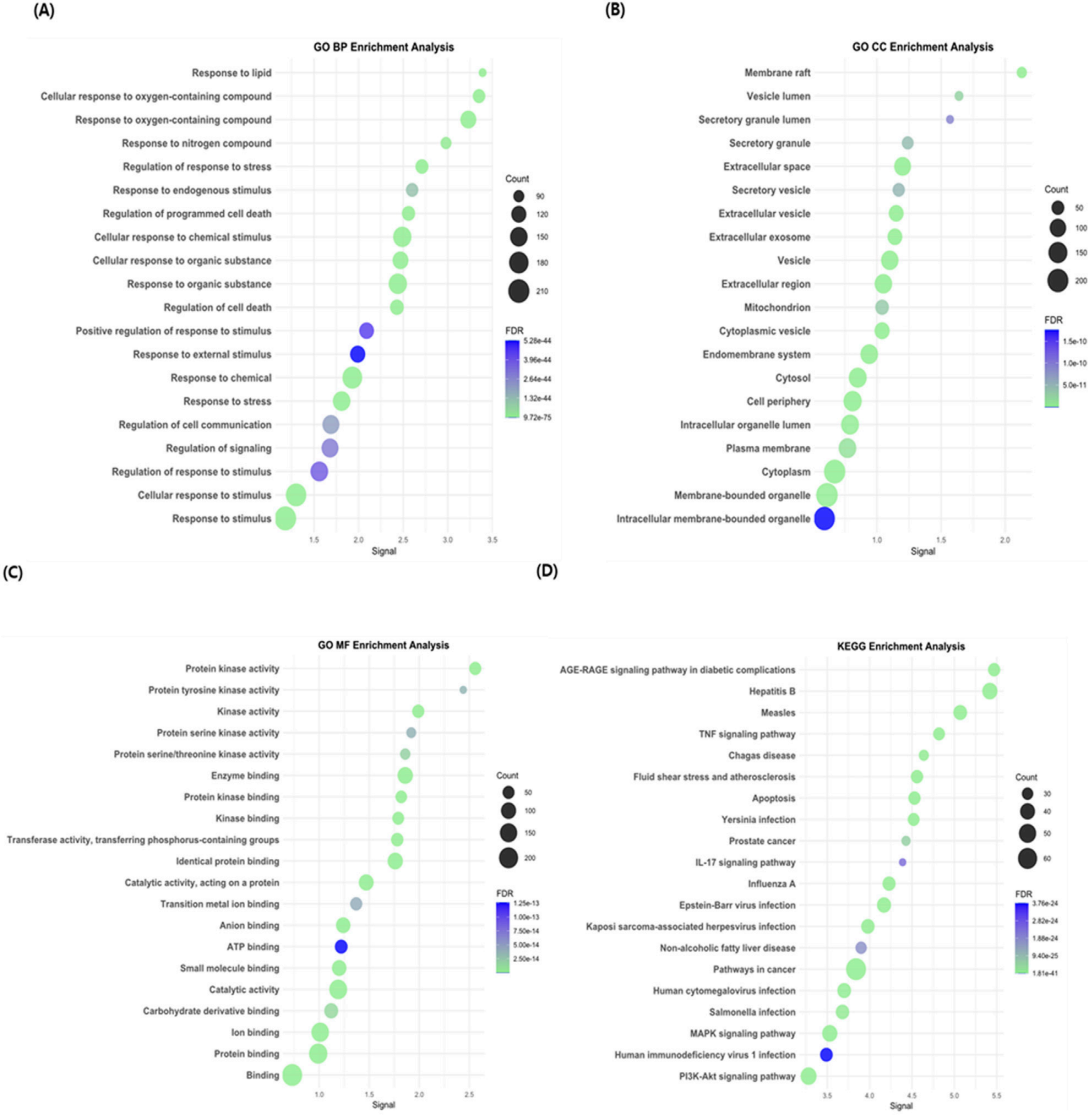
GO and KEGG enrichment analyses of predicted target genes. GO terms are categorized into **(A)** BP, **(B)** CC, and **(C)** MF. GO terms and **(D)** KEGG pathways were enriched using the criteria: FDR <0.01, and gene count ≥5. Dot size indicates the number of genes involved, and color represents the adjusted FDR. Selected terms/pathways related to UC pathophysiology are marked. Terms are ordered by signal values on the x-axis. FDR, false discovery rate; GO, Gene Ontology [BP, biological process; CC, cellular component; MF, molecular function]; KEGG, Kyoto Encyclopedia for Genes and Genomes.

GO and KEGG enrichment analyses were performed to investigate the potential mechanisms by which CAE exerts its therapeutic effects on UC. Significant terms and pathways were identified using the criteria: FDR <0.01 and gene count ≥5. GO analysis yielded 1,344 biological process terms, 94 cellular component terms, and 121 molecular function terms for UC. Additionally, 183 KEGG pathways were significantly enriched. Among these, the top 20 GO terms and KEGG pathways were selected for further analysis. The summary of the results is shown in Figure 3. Notably, KEGG enrichment analysis was used to identify significant enrichment in the TNF signaling pathway, apoptosis, MAPK signaling pathway, PI3K-Akt signaling pathway, and IL-17 signaling pathway, which are known to be involved in UC pathophysiology. Among these pathways, the TNF signaling pathway and apoptosis were most closely associated with inflammation and TJ dysfunction.

#### 3.2.4 Molecular docking analysis

**FIGURE 4:**
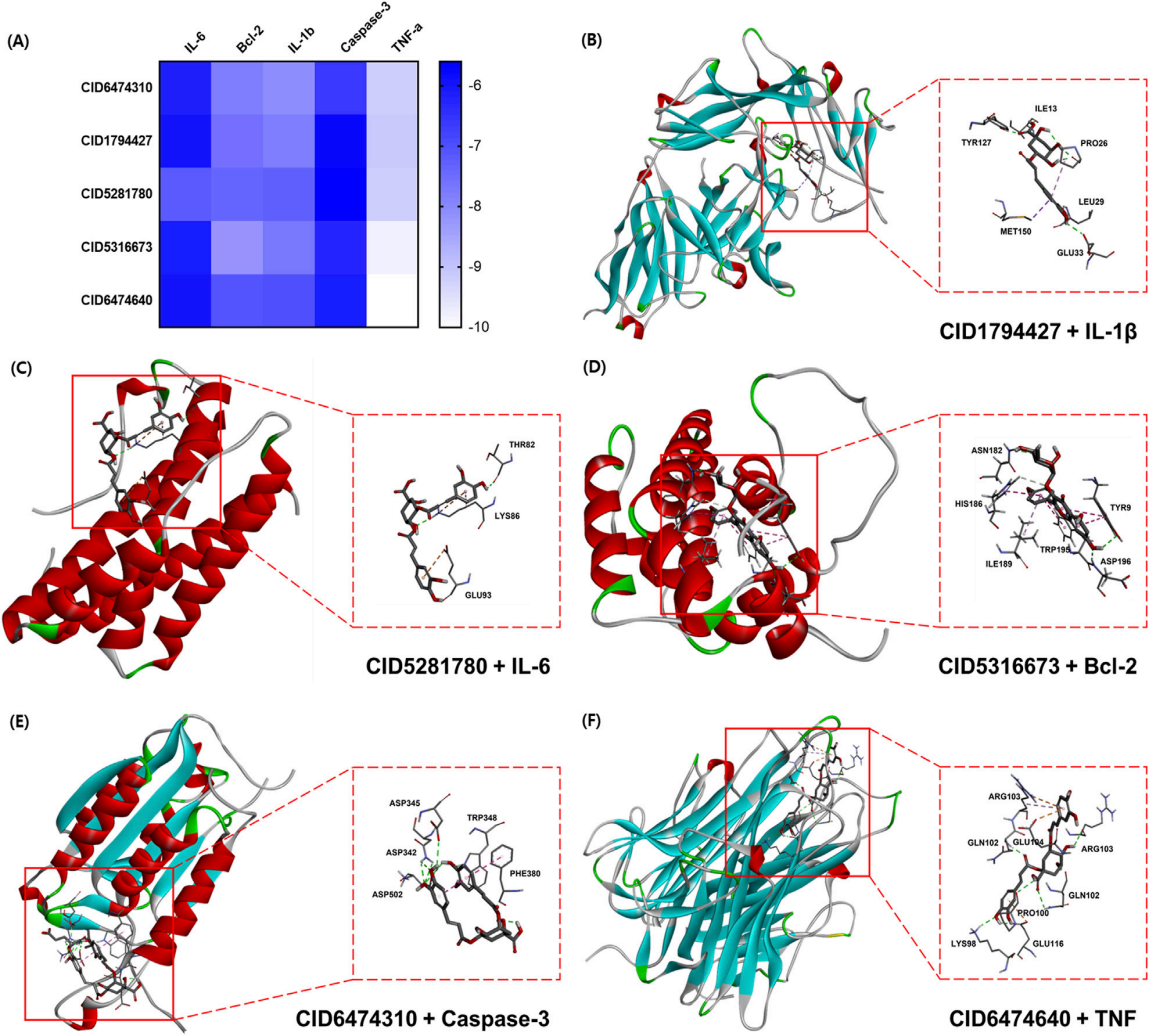
Representative molecular docking conformations of single compounds with their respective target proteins. **(A)** Heatmap of binding energy. Five compound–protein pairs are shown: **(B)** CID1794427 (chlorogenic acid) with IL-1β, **(C)** CID5281780 (4,5-dicaffeoylquinic acid) with interleukin (IL)-6, **(D)** CID5316673 (kaempferol 3-O-rhamnoside) with Bcl-2, **(E)** CID6474310 (3,5-dicaffeoylquinic acid) with caspase-3, and **(F)** CID6474640 (1,3-dicaffeoylquinic acid) with tumor necrosis factor (TNF)-α.

To investigate the therapeutic mechanism of CAE in UC, among the top 10 hub targets of the PPI network, representative biomarkers associated with inflammation and apoptosis, including (PDB ID: 1ITB), IL-6 (PDB ID: 1ALU), Bcl-2 (PDB ID: 1G5M), caspase-3 (PDB ID: 1PAU), and TNF-α (PDB ID: 1TNF) were selected and analyzed through molecular docking with chlorogenic acid (CID1794427), kaempferol 3-O-rhamnoside (CID5316673), 1,3-dicaffeoylquinic acid (CID6474640), 3,5-dicaffeoylquinic acid (CID6474310), and 4,5-dicaffeoylquinic acid (CID5281780) of main components of CAE. The molecular docking binding affinities (kcal/mol) between the five CAE components and the five target proteins are shown as a heatmap (Figure 4A). Each cell represents the relative strength of a protein-compound pair, with color intensity corresponding to the docking score. The results showed that the binding energies between the single compound of CAE and the core target proteins were mostly < −6 kcal/mol, indicating that the predicted CAE active compound had good binding properties with the key targets. Among the 25 available combinations, protein-compound interactions that showed high binding affinity were selected for each protein to build a unique one-to-one mapping (Figures 4B–F). Each complex illustrates the key molecular interactions and binding orientations between the compound and its target protein.

### 3.3 Effects of CAE in DSS-induced colitis mice

**FIGURE 5:**
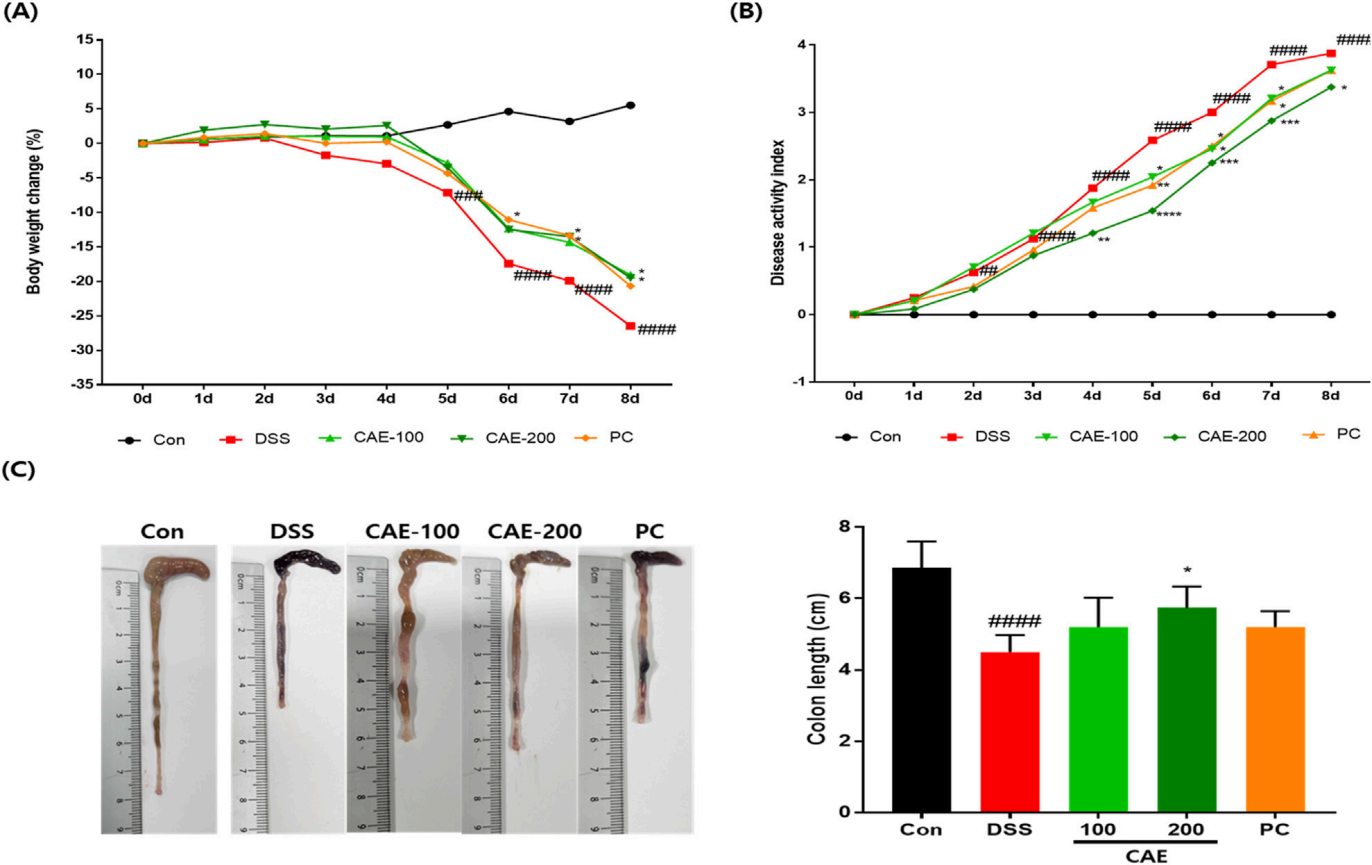
Therapeutic effects of CAE in the DSS-induced colitis model. **(A)** Body weight change, **(B)** DAI, and **(C)** colon length. *##p* < 0.01, and *###p* < 0.001, and *####p* < 0.0001 vs. the control group (Con), *p < 0.05, **p < 0.01, ***p < 0.001, and ****p < 0.0001 vs. the DSS-treated group (DSS). CAE, Carpesium abrotanoides extract; DSS, dextran sulfate sodium.

We induced severe colitis in mice by administering DSS in their drinking water to determine whether CAE improves colitis. Symptoms such as diarrhea, bloody stools, weight loss, colonic shortening, and mucosal ulcers were observed. Excessive inflammatory responses and persistent intestinal dysfunction were caused by DSS-induced weight loss. In DSS-treated mice, diarrhea and rectal bleeding were observed from day 2, and a noticeable change in body weight was observed from day 5. Administration of 100 mg/kg CAE and the positive control mesalamine (PC) ameliorated body weight loss and reduced the disease activity index (DAI), notably 200 mg/kg CAE produced a significantly greater improvement in DSS-treated mice. (Figures 5A,B).

Colonic shortening and luminal surface damage are important clinical manifestations of colitis inflammation (Siegmund, 2002). Colon length was significantly decreased in the DSS group (P < 0.0001) compared with that in the control group, whereas CAE treatment at 200 mg/kg significantly restored colon length (P < 0.05) (Figure 5C).

**FIGURE 6:**
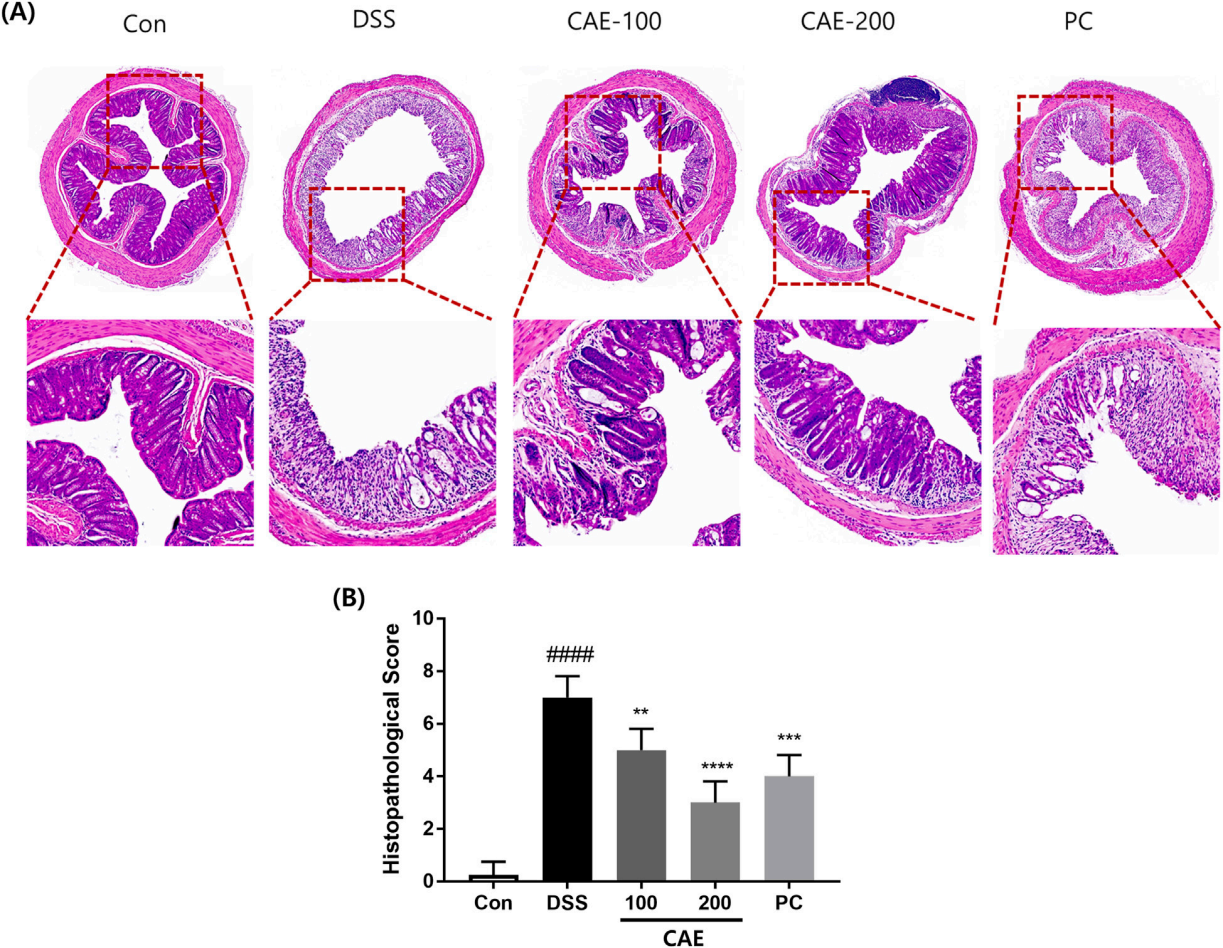
Effect of CAE on histological changes of colon in DSS-induced colitis. **(A)** Representative histopathological images of samples with hematoxylin and eosin (H&E) staining. **(B)** Representative histopathological score. *####p* < 0.0001 versus the control group (Con), **p < 0.01, ***p < 0.001, and ****p < 0.0001 versus the DSS‐treated group (DSS).CAE, *Carpesium abrotanoides* extract; DSS, dextran sulfate sodium.

Furthermore, to assess morphological changes in the colon tissue, H&E staining and semiquantitative analysis of pathological damage to the colon were performed. As shown in Figure 6, colonic distortion of crypt structures, loss of goblet cells, severe epithelial injury, and inflammatory cell infiltration in the mucosal and submucosal layers were observed in the DSS group. However, CAE administration to the DSS-induced mouse model revealed dose-dependent protection of the colon crypt structures and reduced histological inflammation. Notably, 200 mg/kg CAE exhibited a more pronounced protective effect, comparable to or better than the PC, indicating substantial amelioration of colonic injury. These results showed that CAE protected against DSS-induced colitis in mice.

### 3.4 Effects of CAE on intestinal barrier dysfunction in DSS-induced colitis in mice

**FIGURE 7:**
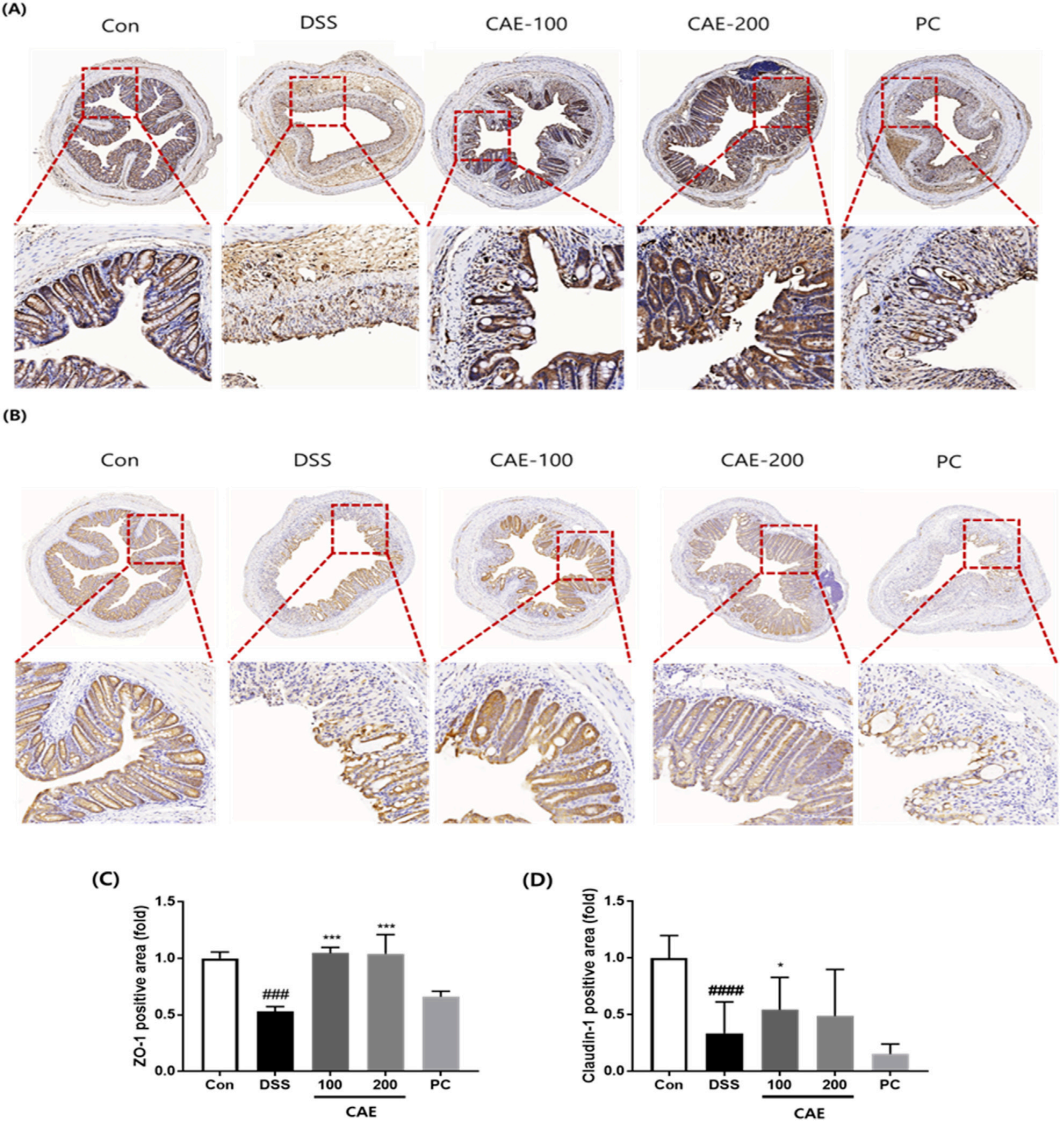
Protective effect of CAE on tight junctions in the mouse colon tissue of a DSS-induced colitis model. Immunohistochemical staining of **(A)** ZO-1 and **(B)** claudin-1 for protein expression, densitometric value of **(C)** ZO-1 and **(D)** claudin-1. ###*p* < 0.001, and ####*p* < 0.0001 vs. the control group (Con); *p < 0.05, and ***p < 0.001 vs. the DSS-treated group (DSS). CAE, Carpesium abrotanoides extract; DSS, dextran sulfate sodium; ZO-1, zonula occludens-1.

Immunohistochemistry analysis was performed to investigate intestinal barrier dysfunction (Figure 7). In the DSS group, ZO-1 and claudin-1 expression notably decreased. However, CAE administration in the DSS-induced mouse model resulted in significantly higher ZO-1 and claudin-1 expression than in mice treated only with DSS. These findings showed that CAE alleviated DSS-induced colitis by protecting the intestinal barrier.

### 3.5 Effects of CAE on the inflammatory cytokines in DSS-induced colitis in mice

**FIGURE 8:**
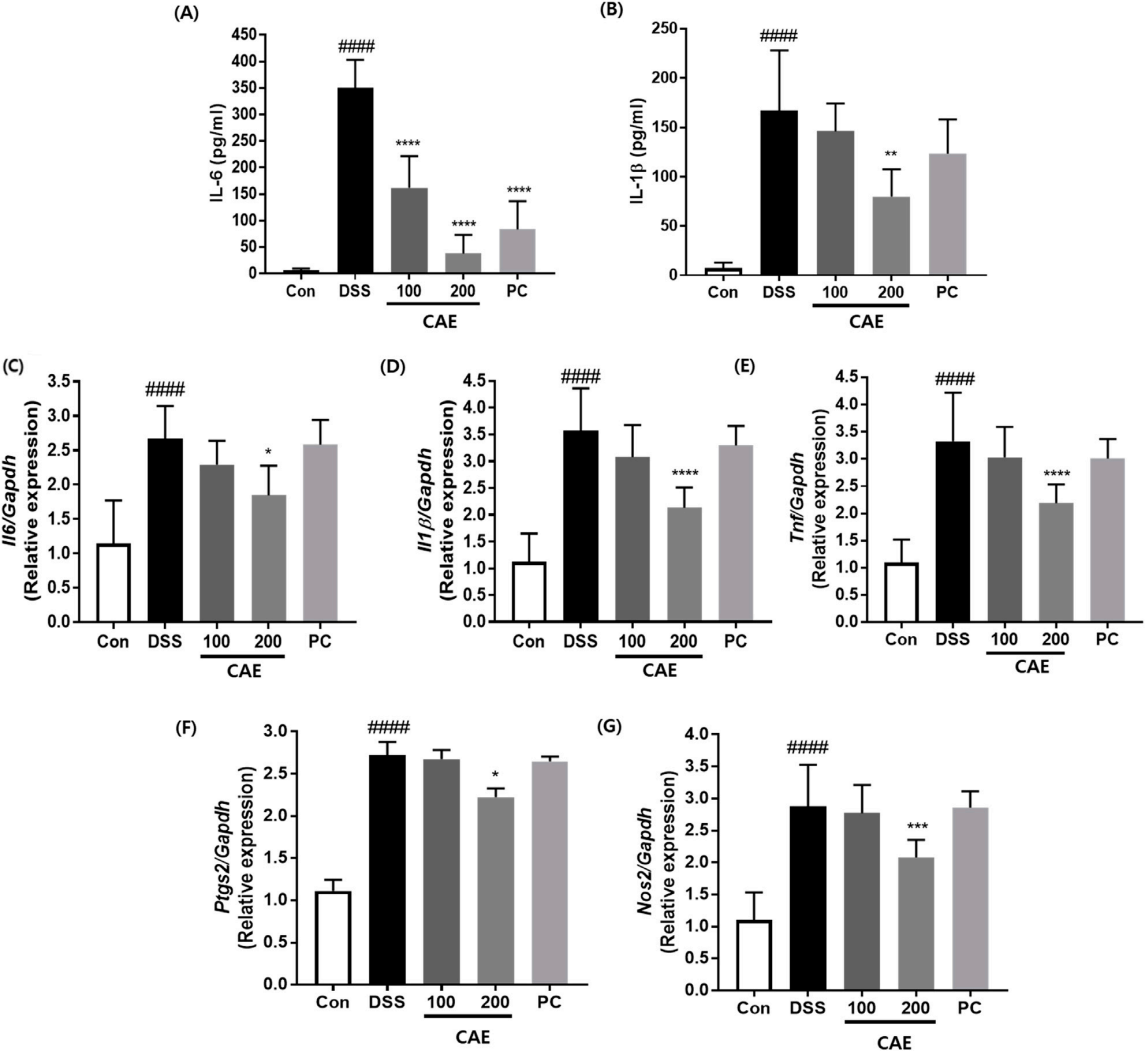
Effects of CAE on pro-inflammatory cytokine levels in DSS-induced acute colitis mice. Serum levels of **(A)** IL-6 and **(B)** IL-1β were measured using an enzyme-linked immunosorbent assay (ELISA) kit. Relative mRNA expression of **(C)** Il6, **(D)** Il1b, **(E)** Tnf, **(F)** Ptgs2, and **(G)** Nos2 in colonic tissue was determined by qPCR. *####p* < 0.0001 vs. the control group (Con); *p < 0.05, **p < 0.01, ***p < 0.001, and ****p < 0.0001 vs. the DSS-treated group (DSS). CAE, Carpesium abrotanoides extract; DSS, dextran sulfate sodium; IL, interleukin; iNOS, inducible nitric oxide synthase; COX-2, cyclooxygenase-2; qPCR, quantitative polymerase chain reaction.

To explore the potential anti-inflammatory activities of CAE in a mouse model of DSS-induced UC, we evaluated the levels of proinflammatory cytokines, IL-6, and IL-1β in the serum. DSS administration significantly elevated the levels of inflammatory cytokines IL-6 (P < 0.0001) and IL-1β (P < 0.0001) in the mouse serum. However, IL-6 and IL-1β levels in mice treated with CAE at 200 mg/kg were reduced compared with those in the DSS-treated group (Figures 8A,B). Real-time PCR was performed to compare the mRNA expression levels of inflammation-related factors in the colon. The mRNA expression of inflammatory cytokines and mediators, including *Il6*, *Il1b*, *Tnf*, *Nos2*, and *Ptgs2*, was significantly higher in the colon tissue of the DSS group than in that of the Con group (P < 0.0001). Both the 100 mg/kg CAE and the positive control (PC) groups exhibited similar reductions, although the differences were not statistically significant. The 100 mg/kg CAE and PC groups showed similar reduction effects, but without significant differences. However, treatment with 200 mg/kg CAE resulted in a significant reduction in the mRNA expression of *Il6* (P < 0.01), *Il1b* (P < 0.0001), *Tnf* (P < 0.0001), *Ptgs2* (P < 0.05), and *Nos2* (P < 0.001) (Figures 8C–G). These results indicate that although DSS treatment increased the expression of inflammatory cytokines in mice, CAE effectively suppressed their expression and attenuated the inflammatory response.

### 3.6 Effects of CAE on apoptosis in DSS-induced colitis mice

**FIGURE 9:**
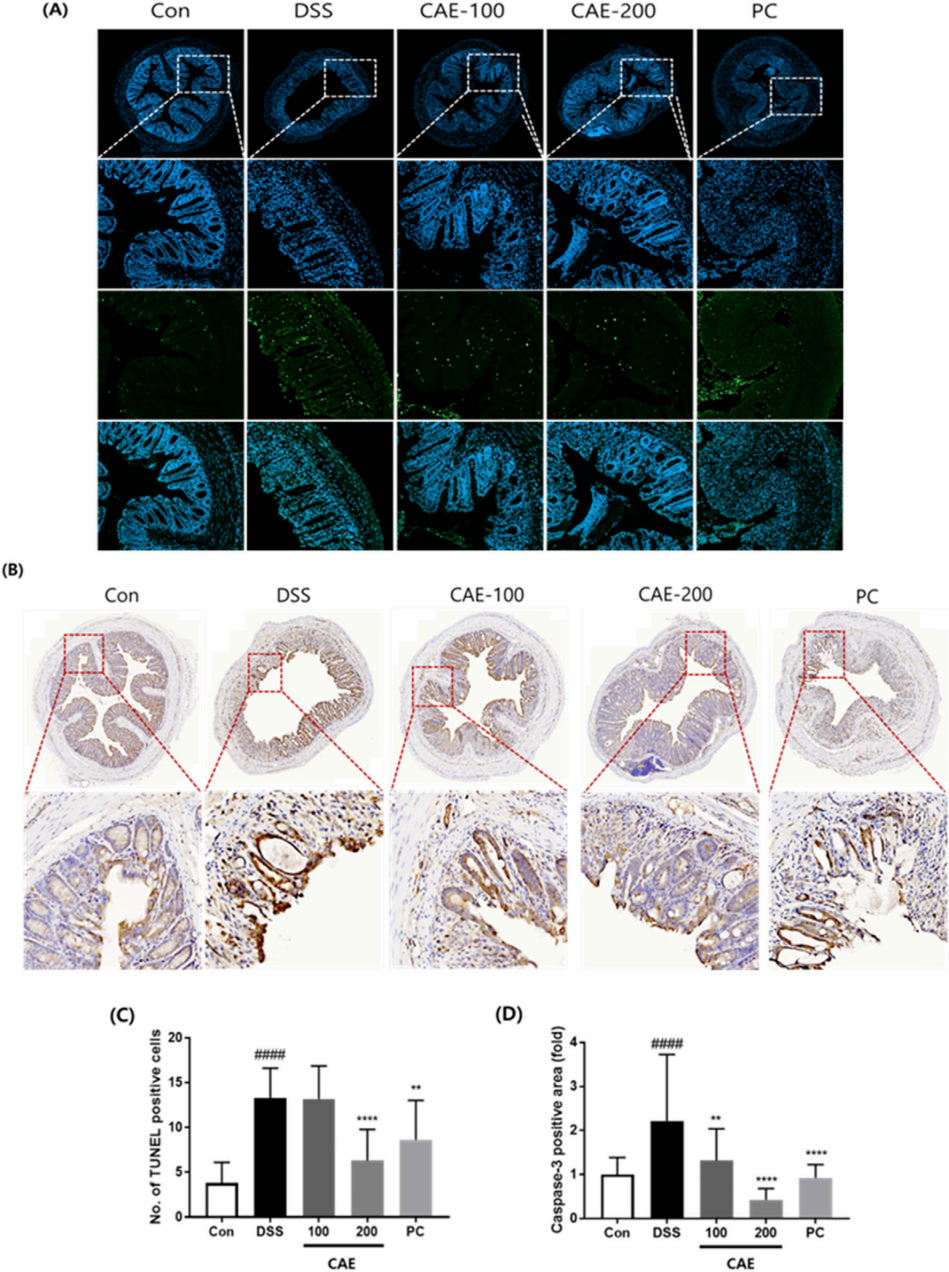
Protective effect of CAE on apoptosis induction in DSS-induced colitis model. **(A)** Representative image of the TUNEL assay, and **(B)** immunohistochemical staining for caspase-3 in colon tissues. Quantitative analysis of **(C)** the number of TUNEL-positive cells, and **(D)** caspase-3. *####p* < 0.0001 vs. the control group (Con); **p < 0.01, and ****p < 0.0001 vs. the DSS-treated group (DSS). CAE, Carpesium abrotanoides extract; DSS, dextran sulfate sodium.

**FIGURE 10:**
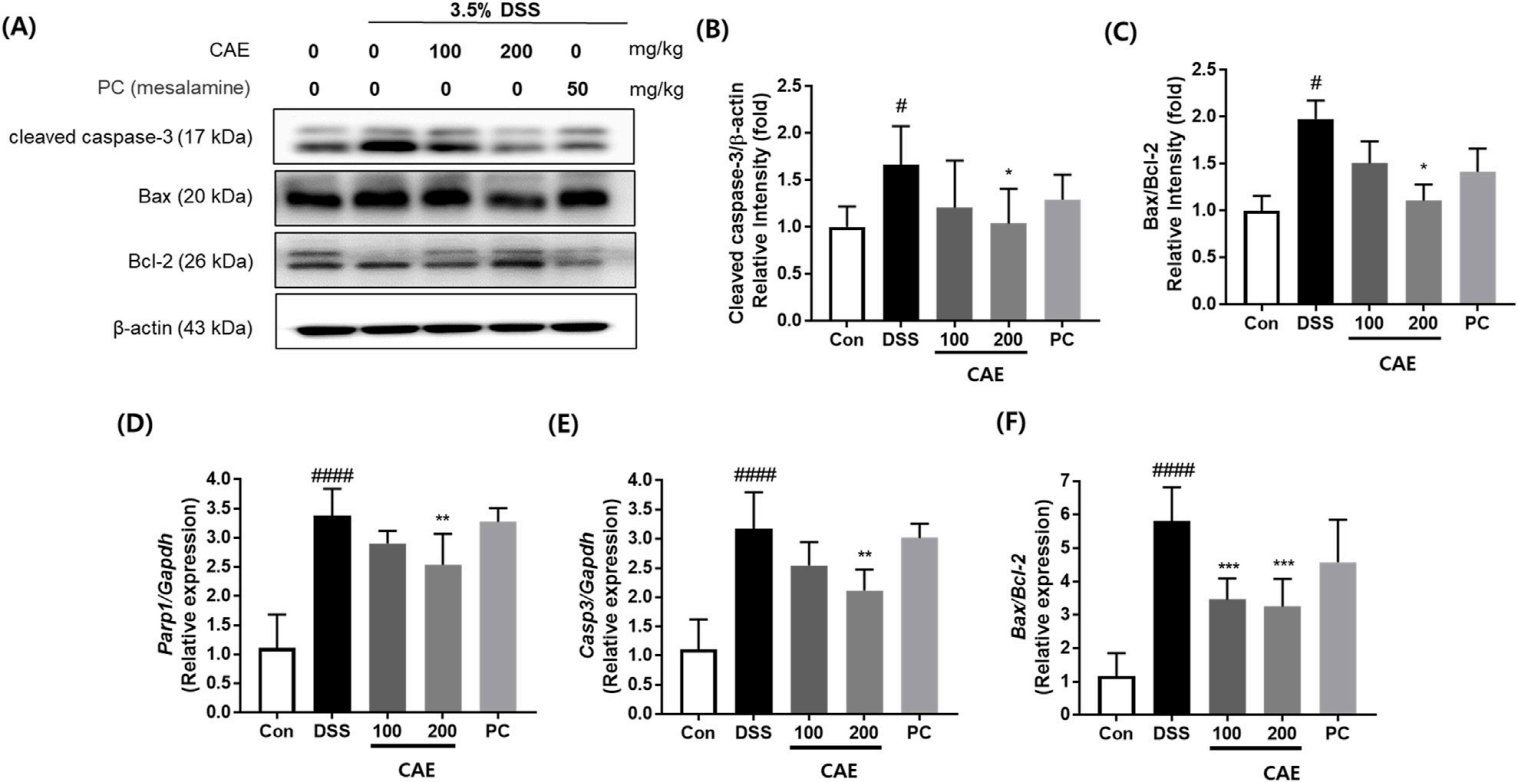
Regulation of protein and mRNA expression levels of apoptosis by CAE in DSS-induced colitis mice. **(A)** Representative expression of caspase-3, Bax, and Bcl-2 proteins was determined using western blotting; β-actin was used as the protein loading control. Expression levels of **(B)** cleaved-caspase-3 and **(C)** Bax/Bcl-2. Relative expression of mRNA of **(D)** Parp1, **(E)** caspase-3, and **(F)** Bax/Bcl-2. *#p* < 0.05, and *####p* < 0.0001 vs. the control group; *p < 0.05, **p < 0.01, and ***p < 0.001 vs. the DSS-treated group (DSS). CAE, Carpesium abrotanoides extract; DSS, dextran sulfate sodium.

TUNEL assay, caspase-3 immunohistochemistry analysis, and Western blotting were performed to examine the changes in apoptosis. In the DSS-treated group, the number of apoptotic cells significantly increased. Conversely, CAE administration significantly decreased the number of apoptotic cells. Reduced caspase-3 expression was observed in the CAE group (Figure 9). Similarly, western blotting and mRNA showed that the expression of apoptosis related marker (caspase-3, PARP, Bax, and Bcl-2) was markedly reduced following CAE administration (Figure 10).

## 4 Discussion

Acute colitis primarily occurs in the large intestine; however, its etiology and pathogenesis remain unclear. The focus of recent research has been on developing candidate drugs that are effective against UC using the multiple and complex components of natural products to simultaneously modulate broad pharmacological activities (Liu et al., 2025; Lu et al., 2023). Natural compounds have been shown to alleviate UC symptoms through various mechanisms, including regulating inflammatory signaling pathways, including NF-κB, JAK/STAT, and MAPK, protection of intestinal epithelial TJs, and restoration of the gut microbiota balance (Gandhi et al., 2023; Gnanasekar et al., 2025; Liu et al., 2021; Long et al., 2025; Subudhi et al., 2024; Wu et al., 2023). In the present study, we aimed to determine the effectiveness of CAE in a DSS-induced colitis model and to explore the therapeutic potential mechanism of CAE using a network pharmacology approach and molecular docking.

First, to identify potential therapeutic targets and pathways of CAE in UC, five CAE components comprising chlorogenic acid, kaempferol 3-O-rhamnoside, 1,3-dicaffeoylquinic acid, 3,5-dicaffeoylquinic acid, and 4,5-dicaffeoylquinic acid, were identified using UPLC analysis. KEGG pathway analysis of these components showed that CAE is closely associated with the TNF pathway, and apoptosis is a key pathway in UC pathogenesis (Eder et al., 2014; Souza et al., 2023) (Figure 3). TNF induces chronic inflammation and cell apoptosis through pro-inflammatory cytokine upregulation. Consistent with this phenomenon, previous studies showed that TNF inhibition attenuates intestinal epithelial cell apoptosis and inflammatory responses in UC mouse models (Garcia-Carbonell et al., 2019; Jung et al., 2019). Therefore, the focus of this study was on the potential of CAE to alleviate UC symptoms by suppressing intestinal barrier dysfunction caused by inflammation and apoptosis, which are among the key mechanisms contributing to UC progression.

DSS-induced colitis in experimental animals is a chemically induced model that is used to mimic the UC pathology in humans. This model has many advantages, including easy use, reproducibility, and the ability to control the extent and severity of inflammation by varying the concentration and duration of DSS treatment, which induces clinical signs of colitis as early as day 1 of treatment. These signs are mainly associated with changes in the expression of TJ proteins, such as ZO-1, occludin, and various claudins (Chassaing et al., 2014; Poritz et al., 2007). Several studies reported that intestinal inflammation is preceded by a decrease in TJ complexes and a subsequent increase in colonic permeability, which, in turn, results in clinical symptoms. These symptoms are observed as typical histological changes in the colon tissue, including a decrease in mucus and goblet cells, epithelial erosion, ulcers, and granulocytic infiltration into the lamina propria and submucosa (Chassaing et al., 2014; Kiesler et al., 2015). Moreover, inflammatory cytokines and mediators, such as IL-1β, IL-6, TNF-α, iNOS, and COX-2, are known to be increased in DSS-induced colitis animal models and are closely associated to developing IBD (Chassaing et al., 2014; Schuerwegh et al., 2003). In our study, clinical symptoms such as diarrhea and bloody stools appeared 2 days after administration of 3.5% DSS and subsequently increased the DAI and shortened the colon, a finding consistent with those found with established biomarkers of UC (Hirai et al., 2010; Wang W. et al., 2025). Moreover, DSS induced a marked reduction in ZO-1 and claudin-1 expression, changes in the colonic crypt structure, loss of goblet cells, severe epithelial damage, and inflammatory cell infiltration into the mucosa and submucosa. Notably, our findings indicate that the CAE administration group showed preserved expression of TJ proteins compared with those of the DSS administration group, showing reduced infiltration of inflammatory cells, and decreased levels of inflammatory mediators. These results show that CAE mitigates colonic inflammation by maintaining intestinal barrier integrity, protecting mucosal structure, and restoring epithelial barrier function by preserving TJs.

Excessive apoptosis in intestinal epithelial cells can damage the intestinal barrier permeability, initiating and exacerbating colitis (Wan et al., 2022). The number of apoptotic epithelial cells increases with the development of UC, leading to the destruction of mucosal integrity and directly affecting intestinal epithelial barrier function (Qiu et al., 2011). Apoptosis is tightly regulated by a balance between pro-apoptotic and anti-apoptotic proteins, and the most representative molecular markers involved are Bax, Bcl-2, and PARP (Han et al., 2022). PARP-1, a key member of the PARP family, regulates multiple cellular functions, including chromatin remodeling, DNA repair, and cell death (Sethy and Kundu, 2022). During apoptosis, PARP is cleaved by caspases, especially caspase-3; the presence of cleaved PARP is a hallmark of apoptosis (Jeon et al., 2023). Bax and Bcl-2 work antagonistically; Bax promotes cell death, while Bcl-2 inhibits it (Wang M. et al., 2025). Hence, upregulating Bax and PARP-1 and downregulating Bcl-2 may be important indicators of apoptosis. The results of this study show that CAE exerts anti-apoptotic effects in DSS-induced UC by downregulating pro-apoptotic proteins, including Bax, Cleaved-caspase-3, and PARP and upregulating the anti-apoptotic protein Bcl-2.

The main CAE components include chlorogenic acid, kaempferol, 3-O-rhamnoside, 1,3-dicaffeoylquinic acid, 3,5-dicaffeoylquinic acid, and 4,5-dicaffeoylquinic acid. In a previous study, chlorogenic acid was reported to have antioxidant and anti-inflammatory effects and promote the growth of beneficial intestinal bacteria while inhibiting the growth of harmful bacteria in DSS-induced colitis (Xu et al., 2024). Reportedly, dicaffeoylquinic acid, which contains 3,5-dicaffeoylquinic acid and 4,5-dicaffeoylquinic acid, alleviates DSS-induced colitis, potentially by modulating alterations in the gut microbiota associated with the disease (Wan et al., 2019). Meanwhile, the molecular docking results showed that these main components of CAE bind effectively to targets, including TNF, IL-6, IL-1β, caspase-3, and Bcl-2, which are critical in the inflammatory cascade underlying UC. Specifically, pro-inflammatory cytokines (TNF, IL-6, and IL-1β) indirectly activate endothelial cells through inflammation in target cells. CASP3 and BCL2 play critical roles in apoptosis and survival, which are central to UC pathophysiology. However, the doses of these compounds (chlorogenic acid, 3,5-dicaffeoylquinic acid, and 4,5-dicaffeoylquinic acid) used to evaluate their efficacy were significantly higher than those of CAE, making it difficult to attribute the effects of CAE solely to the actions of the individual compounds. Furthermore, the anti-inflammatory effects of kaempferol 3-O-rhamnoside and 1,3-dicaffeoylquinic acid have been reported; however, their effects on UC have not been studied (Hufnagel et al., 2024; Zhao et al., 2021). Therefore, further studies are required to determine whether kaempferol 3-O-rhamnoside and 1,3-dicaffeoylquinic acid are the active components of CAE or whether the effect is due to a synergistic effect among other compounds present in CAE.

## 5 Conclusion

Our findings show that CAE effectively alleviates UC symptoms by preserving TJ protein expression, reducing inflammatory mediator levels, and restoring epithelial barrier integrity through anti-inflammatory and anti-apoptotic mechanisms. Thus, CAE is a promising therapeutic option for UC.

## Data Availability

The original contributions presented in the study are included in the article/Supplementary Material, further inquiries can be directed to the corresponding author.

## Abbreviations

Bcl-2, B cell lymphoma 2; BP, biological process; CAE, *Carpesium abrotanoides* extract; CC, cellular component; COX, cyclooxygenase; DAI, disease activity index; DSS, dextran sulfate sodium; FDR, false discovery rate; GO, Gene Ontology; IBD, Inflammatory bowel disease; IL, interleukin; iNOS, inducible nitric oxide synthase; KEGG, Kyoto Encyclopedia of Genes and Genomes; KIOM, Korea Institute of Oriental Medicine; MF, molecular function; OMIM, Online Mendelian Inheritance in Man; PC, positive control; PCR, polymerase chain reaction; PDB, Protein Data Bank; PPI, protein–protein interaction; TJ, tight junction; TTD, Therapeutic Target Database; TUNEL, transferase dUTP Nick End Labeling; UC, ulcerative colitis; UPLC, ultra performance liquid chromatography; ZO-1, zonula occludens-1.
